# Electronic Medical Records in Greece and Oman: A Professional’s Evaluation of Structure and Value

**DOI:** 10.3390/ijerph15061137

**Published:** 2018-06-01

**Authors:** Ourania Koutzampasopoulou Xanthidou, Liyana Shuib, Dimitrios Xanthidis, David Nicholas

**Affiliations:** 1 Department of Information Systems, Faculty of Computer Science and Information Technology, University of Malaya, Kuala Lumpur 50603, Malaysia; 2 Management Information Systems, Sohar University, Sohar 311, Oman; dxanthidis@soharuni.edu.om; 3 CIBER Research Ltd., Newbury RG14 7RU, UK; xanthidisdim@gmail.com (D.X.); Dave.Nicholas@ciber-research.eu (D.N.)

**Keywords:** Electronic Medical Record (EMR), demographics, contents and structure, economic feasibility, qualitative study, Entity Relationship Diagram (ERD)

## Abstract

An Electronic Medical Record (EMR) is a patient’s database record that can be transmitted securely. There are a diversity of EMR systems for different medical units to choose from. The structure and value of these systems is the focus of this qualitative study, from a medical professional’s standpoint, as well as its economic value and whether it should be shared between health organizations. The study took place in the natural setting of the medical units’ environments. A purposive sample of 40 professionals in Greece and Oman, was interviewed. The study suggests that: (1) The demographics of the EMR should be divided in categories, not all of them accessible and/or visible by all; (2) The EMR system should follow an open architecture so that more categories and subcategories can be added as needed and following a possible business plan (ERD is suggested); (3) The EMR should be implemented gradually bearing in mind both medical and financial concerns; (4) Sharing should be a patient’s decision as the owner of the record. Reaching a certain level of maturity of its implementation and utilization, it is useful to seek the professionals’ assessment on the structure and value of such a system.

## 1. Introduction

An EMR is defined as an electronic record of health-related information on an individual that is created, gathered, managed, and consulted by authorized clinicians, medical units’ professionals of various types and staff [1,2,3]. During the last decade or so, medical units are gradually transitioning from the traditional paper medical health records to Electronic Medical Records (EMRs) [4,5], but they are not always online so as to share the medical files for the same patient. The diversification of standards by the different medical units makes it difficult to design a global EMR and Electronic Prescription System (EPS) [6]. 

The Institute of Medicine reports that medical errors may be responsible for 44.000 to 98.000 deaths per year in the U.S.A. alone [5]. Therefore, it is essential to improve the quality of healthcare services as well as its security by establishing the requirements to implement the EMR. The U.S. Department of Health and Human Services (HHS) with the Health Information Technology for Economic and Clinical Health (HITECH) have a list with the ‘Certified Health IT products’ which are tested and certified by the Office of the National Coordinator for Health IT (ONC). This list contains some 3990 products many of which do not exist anymore or are older versions but, still, it includes more than 1000 certified programs [7]. 

**Figure 1:**
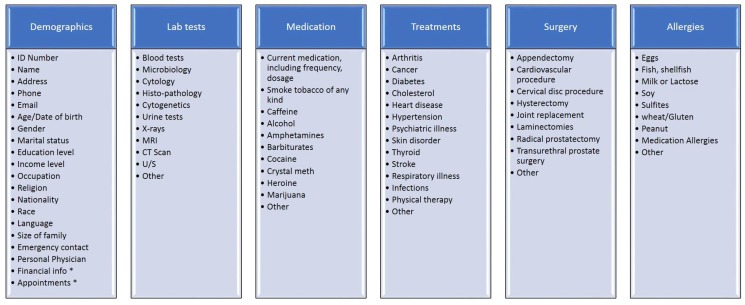
A Contents and structure of EMR system.

To achieve a meaningful use, one of the requirements is to capture demographic information such as name, address, gender, age, race, etc. [8]. Figure 1 illustrates, among other things, most of these demographics as they appear in one of the EMR systems available on the market [9]. Such a system is the ‘FreeMED’ which for each patient’s record use almost all the demographics but in different levels, i.e., demographics (title, name, gender, date of birth), address, contact (preferred contact, phones, email address), personal info (marital status, employment status, SSN, race, religion, language, etc.), medical info (primary facility, preferred pharmacy, MD’s name, etc.), coverage info (insurance information) and authorizations schemes [10]. Other similar software products follow more or less the same structure for an EMR [11,12,13,14,15,16,17,18].

Given the variety of systems available to choose from, interoperability looks like not a realistic scenario and there are a lot of relevant issues to be addressed [19]. Hence, though ideally all these systems should be able to function in an integrated way but that is not the case yet. Another concern, especially for medical doctors (MDs from now on) and nursing practitioners, is the complaint that a lot of time is wasted to input the data of a patient or even to read a file instead of practicing medicine [3]. 

Another issue associated with the implementation and support of such an EMR system is its economic feasibility. There were such efforts in U.K. and in France, among other countries, that were abandoned as not having good prospects or were decided to be planned and repeated for better results [20,21]. Indeed, given the financial constraints in the medical organizations, which do not allow their management to invest without limits on EMR systems, and keeping in mind the various technical concerns that need to be addressed when developing such a system, e.g., network security and availability, scaling of the database, training the personnel, etc., it makes it a must to develop and communicate a strategy of the role and value of such a system in the organization and prepare at least a basic business plan, a road map, of how to achieve this. This, in turn and gradually in time, will facilitate, if deemed necessary and/or useful, the sharing of such a record with other medical organizations [22,23,24].

Finally, there is the concept of the medical card, which stores partial information of a person such as name, identification (ID) and age, and is based on the magnetic swipe technology [25]. It is assumed to have the size of a credit card and include medical data such as patient’s information, the history of present and previous illness, family or social history, etc. It can be in magnetic form or based on the optical recognition system and readable by a peripheral reader [26]. The stored information is expected to be encrypted for security and privacy issues to avoid malpractice by unauthorized users. It is ready to be updated with new treatments, observations or any other new data. Using such a medical card the patients, especially those with certain diseases, may carry all their medical history on them always. In cases when the patient is unable to communicate, unconscious, or doesn’t know enough about his/her problem and treatment this medical card might be the only solution to the problem of providing the patient’s history to the medical staff when needed. In an effort to save the life of such people, the health professionals ask for very expensive lab tests when time is of critical importance and the medical card may be the answer to act fast and avoid unnecessary costs [27,28]. If properly planned, this concept and mechanism could be integrated in an EMR system for the benefit of both the medical units and the patients.

There have been several previous surveys on the structure and possible elements of an EMR system from a health consumer’s viewpoint [29] but it is difficult to find studies focusing on the evaluation of such systems from the medical professionals’ stance. This study is an effort to bridge this gap of knowledge.

## 2. Methods

This is a phenomenological qualitative study [30] that aims to explore the experiences of the various health professionals with regard to the contents and structure of an EMR, the economic issues associated with it, and whether such a record should be shared between health professionals and health organizations. It took place in the natural setting of the health units’ environment (i.e., in the hospitals).

### 2.1. Instrument

The instrument, i.e., the interview schedule, was prepared by the researchers following the current guidelines of qualitative research [30]. The topics/themes and investigative questions/sub-questions under discussion were chosen in such a way as to ensure that triangulation was achieved when validating the contents of the instrument [31,32]:

- They came from a review of the literature [33],
- The interview schedule was moderated by five academics from different countries and universities who gave their consent on its construct validity,
- After the moderation, six pilot interviews took place one from each of the different types of experts in the two countries, Greece and Oman. During these pilot interviews [34], after every research and investigative question, the interviewee was asked a question on the content validity. The question was: “How would you evaluate the question as to its relation to the topic discussed? (i) Not relevant; (ii) Important but not essential and (iii) Essential. The pilot interviews did not result in any new points to be included in the interviews.

As far as the reliability of the instrument (interview schedule), it was secured by the highly rigorous data collection procedure followed which is explained in detail in the next section.

Five (5) different open-ended questions were asked during the interviews and the participants were welcomed to elaborate their thoughts:

1. What are the basic demographics that must be included in a patient’s record? What do you think about the suggestions of the Figure 1? [2,8,35]
2. Lab tests, diagnostic images, treatments, therapies, drugs administered, patients identifying information, legal permissions and allergies (as they appear in Figure 1) are possible elements to be included in the EMR [2,19,22,36]. What is your view on this?
3. Some experts believe that these structure and contents of the EMR will reduce the repetition, save time and cut costs if they are available on a patient’s record [1,2,37,38]. What is your stance on this matter?
4. How do you think that the size and depth of the data to be included in the EMR will affect the cost and, eventually, the decision to implement and support such a system? [1,37,38]
5. Do/would you share EMR records of your patient’s with other organizations? [2,19]

During the interviews, the participants were given the Figure 1 above and as many explanations as they needed to understand the issues. Finally, it should be noted that, since the study was affiliated with the University of Malaya (Kuala Lumpur, Malaysia), special permission was requested from the university authorities and was granted to the researchers in the form of a cover letter addressed to the prospect participants and duly signed by those authorities [30].

### 2.2. Sample

The geographic scope of this research are the two countries Greece (developed) and Oman (developing) [39]. These countries have dramatically different cultures and religious backgrounds which would make it interesting to see the various approaches on the subject [40]. These countries were selected for two purposes. One, there was an expressed interest by the high-ranking management of the major hospitals in these countries that stressed the need of their organization for such a research. Two, it was feasible to conduct the in-depth interviews in-person due to close proximity of most of the hospitals to the researchers. Hence, the authors achieved, even though in a limited way, their goal to study a European Union (Eurozone) country and a GCC Muslim country.

In order to secure a holistic account of multiple perspectives, coming from different professionals associated with the health sector, a balanced purposive sample of these different individuals was selected [30] to ensure triangulation of the data collected. They had to be as highly ranked as possible to be able to provide deep insight and experience on the questions asked in the context of their particular role in their health organization [41]. These high-ranking professionals were (from each of the health organizations): (a) An administrator and a legal advisor or quality assurance person; (b) A medical doctor and a nursing practitioner; and (c) A technical person either from the lab or from the pharmacy and an ICT expert. The patients were not part of the study and neither were the general public as explained before due to the nature of the study (i.e., qualitative and not quantitative). 

The sample was purposive because it was necessary to seek, find, and approach these professionals [30,42,43]. For this reason, a number of large medical organizations were contacted, both in Greece and Oman, to try and reach the CEOs or highest administrator officers in these units. The purpose was two-fold, i.e., to get these individuals’ permission to conduct the interviews in their hospitals and, if possible, to interview even them. An effort was made to conduct a balanced (not necessarily equal) number of interviews from the two different countries, from the public and the private sector and from the different professions. The results were as follows:

1. Greece: 26 (four hospitals); Oman: 14 (two hospitals),
2. Public: 20; Private: 20,
3. CEO/Admins: 7; MDs: 7; Nurses: 7; Lab/pharmacists: 7; ICTs: 5; Legal/lawyer: 7.

### 2.3. Data Collection

The data collection process started in 5 June 2016 and was completed on 22 July 2016. During that period forty (40) interviews were conducted, 32 of them in person in the natural setting of the interviewees’ health environment, i.e., in the health unit itself [30], and eight were conducted online (via email) [44] because of technical difficulties related to the distance from the health units. Since this was a qualitative study the researchers conducted the interviews themselves [30]. The data collection process was as follows for the in-person interviews:

- An introductory letter was handed to the management of the health organizations at the highest possible level to obtain permission to conduct the interviews with the selected professionals in the health unit [30].
- The same introductory letter was given to the interviewees explaining the study, its purpose and the organization it originated from in the form of a cover letter with the logo of the affiliated university [30].
- The researchers explained the procedure of the study step by step, through an invitation letter.
- The interviewees were asked (and all of them agreed) to sign a letter of intent which included their name, position, organization, phone, email, signature, date and, when available, stamp, given that their privacy and confidentiality will be kept all through the process [30].
- The researchers asked the interviewees for permission to audio record the conversation and all of them gave their consent except for those under the email scheme in which case that was not necessary [30].
- The transcript of each of the audio recording was emailed to each of the interviewees to confirm it or note possible disagreements. This was part of the strategy followed to ensure the validity of process [30,45].
- The findings were confirmed both by the participants and by eight peer reviewers who provided their feedback as part of the validity process [30,45].

The average time of the interviews was 40′00″, with a minimum of 17′21″ and a maximum of 1 h 17′43″. For the online interviews the process was the same but was conducted through emails and without audio recordings. In Section 3 where quotes are given they belong to the interviewees themselves. In every stage of the procedure above the researchers made a conscious effort to avoid “taking sides” on the themes discussed by the interviewees providing only explanations of the terms when necessary [30]. It should be noted, though, that the participants were fully aware of the information systems stance of the researchers all through the process. (Evidence of all the steps above and their outcomes are available upon request given assurance of the confidentiality necessary as part of the code of ethics rules and roles).

## 3. Results

### 3.1. Contents and Structure of the EMR

The interviewees were asked to confirm the validity of the EMR contents and structure as the one suggested to them (Figure 1). Their responses elucidate that the task of forming a structure approved by all different types of health professionals is insatiable. The most significant statements of their responses appear in Table 1.

There are a few points the majority tend to corroborate. One is that almost all confirm the contents of the EMR as suggested in Figure 1 with some variations mainly in the labels of the categories and some of the contents, e.g., they pointed that instead of the label “treatments” the particular category should be labeled “diagnosis” as treatment comes at a later stage than the diagnosis. Another is that the vast majority support the practice of having the contents of the EMR in different levels not all accessible by all the various types of health professionals. A third point is the professionals’ stance that it does not help to ask one’s personal income for the EMR since whatever financial information is necessary, e.g., how the diagnosis and treatment costs will be covered, this will indirectly but clearly and concisely appear in the insurance record of the patient. Finally, almost all stressed the need to follow an open architecture, i.e., an EMR model open to changes to its contents (mainly additions of subcategories).

There are several other issues, though, which are highly controversial especially seen from the different professionals’ viewpoints. Some believe that although it might seem racist to ask a patient’s race, however it is often crucial in order to conclude on the correct diagnosis and select the appropriate treatment since there are certain health issues closely associated with particular races of people. Others completely reject this idea without discussion. Some believe that religion should be included in the patient’s record to help understand the patient’s habits and, possibly, culture that affect an individual’s health condition while others find such an idea highly disputable, to say the least, pointing to the discrimination practices that it might lead to. Likewise, with the cases of a person’s nationality, language, and religion; some find the idea of their inclusion to the EMR useful or even essential while others find it unacceptable.

Finally, there were some interesting suggestions of elements that should be included in the EMR like a particular category of ‘eating habits’ or ‘life style’. This kind of information, i.e., smoking, night life, special nutrition, vitamins etc., sometimes, is very important since these ‘habits’ have different interactions with some treatments. Given these, the MD could provide better health care to the patients and avoid mistakes that are related to the elimination of the effects of a medicine because of these behaviors. 

**Figure 2:**
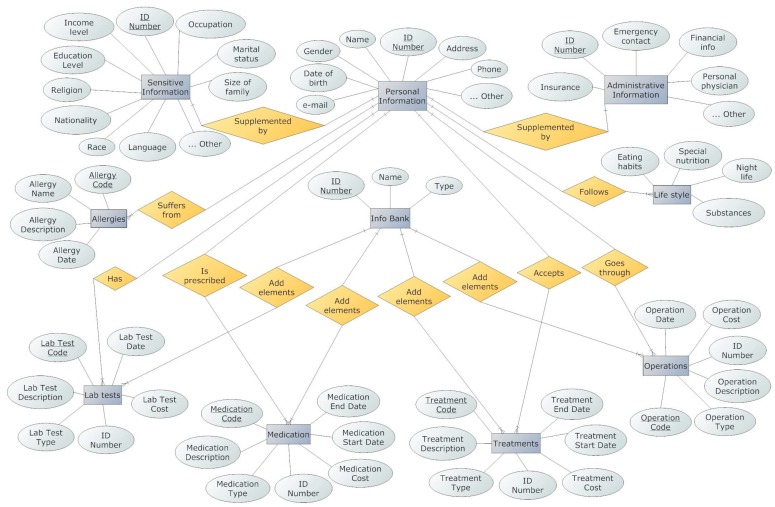
A suggestion of an Entity Relationship Diagram for the EMR system.

Figure 2 illustrates an Entity Relationship Diagram of an EMR that follows the professionals’ suggestions.

### 3.2. Economic Feasibility

The professionals were also asked to discuss the effect of such a structured EMR system, like the suggested one, on time and costs savings possibly through avoiding or reducing unwanted and unnecessary redundancies of medical exams. Moreover, they were asked to discuss on the economic feasibility of implementing and supporting such a system based on its projected size and depth. 

In general, the participants were mainly positive to the idea of realizing such a system because it would be beneficial for the patients and their health. The MDs, in particular, agreed this is above money or any other concerns and issues. Only some of them underlined certain conditions to be satisfied before such an effort is initiated. The main point stressed was that such a system should be gradually developed, since it will be for the benefit of the patients, but a plan should be formed as to the stages and depths of each development increment. 

One of the most serious concerns, coming from the MDs in particular, is that it often leads to wasting time in front of a screen entering or reading data instead of practicing medicine. Another important point is that, though such a system is beneficial for the patients, however, there are other serious categories of expenditure that a government has to, also, cover and, hence, in times of recession such as the ones Greece and Oman are both facing, it would be wise to plan for such system in light of the larger picture of the countries’ economies. Their most significant statements are given in Table 2.

### 3.3. Sharing Medical Records

The professionals were asked if they would share the patient’s medical record with other medical organizations. Their most significant statements appear in Table 3.

The general trend is that the health professionals are positive to this practice as long as it secures the privacy of the patient and sharing takes place only between certified medical practitioners (MDs and nurses) in certified medical units. Some of them stress the fact that, since the EMR belongs to the patients, it is their legal right to have it shared between health organizations and their professionals. In fact, some believe that nowadays it is more necessary than before given the extensive mobility of a significant part of the global population as it is evident from the growth of the tourism industry internationally and the demand for professionals to move around the world more frequently and for extensive periods of time than before.

This trend is not significantly different between Greek and Omani professionals, neither it is between those in the public or private sector or the types of professionals. It only appears that those with more than 10 years of professional experience tend to be more “loose” with the need for authorization schemas and protocols to be followed in such cases.

### 3.4. Limitations

The main limitations of the study are related to the countries it took place, the language used, and the fact that only medical unit professionals were interviewed and not those from higher organizations e.g., ministries, health insurance companies. The study took place in Greece and Oman. Ideally, it should take place in the U.S.A., E.U., China, India, and the Arab World, including Greece and Oman. However, given the time constraints and budget available, this would be practically infeasible. New research efforts could cover this issue. 

One of the problems we faced, especially in Oman, was that many professionals either could not speak English or their language command was poor. This may have resulted into problems of understanding completely the questions of the interviews which, by nature, where difficult and deep. Hence, it would be more appropriate to have Arab translators to help during the interviews or well-trained Arabs to conduct them. If a new study, targeting only the Arab world, is performed this problem would be addressed. Additionally, ICTs were difficult to reach and, hence, difficult to interview. Same with the lawyers who did not want to participate and when they did they were not very open to describe their views as they were worried about the confidentiality of their interviews.

## 4. Conclusions

This study resulted in several conclusions regarding the contents and structure of the EMR, its economic value and whether such a record should be shared between health professionals and organizations. 

First, as far as the demographics are concerned, they should be divided into different categories not all of them accessible and/or visible by all users of the EMR. The most sensitive information would not appear in the first level and the users would not have to spend time to go through all of it. The elements of race, religion, income level and education level should be in the sensitive information category, or not at all. Some professionals insist in further including a particular category like ‘eating habits’ or ‘life style’. Most of them suggest that the label of the “treatments” category should be replaced by “diagnosis” as better reflecting its elements.

Second, all the interviewees agreed it would be better if the implementation of such a structure is following an open architecture, i.e., the medical units can start with something smaller, probably in a basic level, check it, evaluate it, and gradually develop a larger system. This is mainly because of the difficulties the health organizations, like many others, are facing during these times of economic recession in both countries of Greece and Oman. Since the implementation of such a system needs a serious budget, the management people cannot overlook or underestimate the financial issues. Hence, although indeed the main argument is that saving human lives and/or treating people, regardless of their personal, financial or other situation, is above money, however, money is not limitless. When, almost always, there are budgetary constraints for running a medical unit, there is an unfortunate demand to limit spending in certain categories of expenses as much as that is possible.

Third and on the bright side, even in developing countries, where the average income is usually low compared to the developed E.U. countries, the U.S. and some others, and one would assume that health care costs would be a decisive factor for the health consumers to bear, it is worth noting that, still, these costs are not an obstacle for the consumers to ask for quality health care service even in considerably high, for them, rates as studies show [46]. That could imply that the managers of the health care units should be more interested in ensuring the quality of the services they provide rather than the costs of it. A properly implemented and supported EMR system would be an important factor contributing to such quality service even if that means seeking for public-private partnerships to overcome such financial obstacles [47]. Recent E.U. statistics show that, despite the financial risks, investment in health and eHealth has good potential and possible losses are estimated at a low of just 1.4% of the GDP in Europe. Hence, investing in good quality health care services, including proper EMR infrastructure has very good prospects for returns [48].

As to the practice of sharing the medical records, most of the professionals agreed it should be done either under authorization or even without. The idea of sharing would help not only the patient but the international research efforts since it could provide valuable statistics. Special attention should be given, though, so that there is no sharing of patients but just their medical data. However, it should be noted that there is a very thin line that separates one from the other and, often, it is a matter of interpretations. Eventually, no matter if the patient’s medical record is in a medical unit system or in a national one, it always belongs to the patient him/herself and s/he is responsible to make these decisions. 

Such a system is a central piece of a modern health care unit especially in this era of cross-border health care in which individuals from different countries with health care inequalities try to take advantage of those in an effort to save either time or costs in covering their health care needs. Particular example of this is the case of Poland and Germany in which individuals from Poland visit and receive health care from German health units to save time and, on the other hand, others from Germany visit Polish hospitals to receive health treatment at much lower costs [49]. Without a proper EMR system, implemented and controlled not only in a local but also in a country and an international scale, the cross-border health care will cause severe problems to the whole National Health Systems of various countries [50].

Finally, clearly, according to the medical professionals organizing and implementing an EMR is for the benefit of the health consumer and the health unit, since, it might lead to significant savings in time and costs by avoiding medical and other test redundancies. However, the governments can also benefit from such systems deployed in the public and private hospitals of their responsibility since EMR deployment everywhere has the potential of dramatically reducing the workload of the medical professionals at times when there appears to be a certain shortage of those especially in certain European but also in the GCC countries and elsewhere [51]. 

It goes without saying that there is a move towards eHealth services, i.e., providing different types of health services over the Internet, on a global scale. Such services might range from simply making appointments online to have various types of health consultancies online when and where that is possible. The EMR certainly helps in facilitating such systems if the appropriate Information and Communication Technology infrastructure is in place, which, though, is not always a given even in advanced regions like in some European Union countries [52]. We are in the information age when almost every human-related activity is attempted to be managed online. Health-related activities are not different from this general global paradigm despite some hesitation from a part of the health professionals [53].

## Figures and Tables

**Table 1:** Contents and structure of the EMR—significant statements.

Alias, Position, Country, Hospital Type	Quotes
P7, M.D., Greece, Private	“Race: It is important. For example, there are medicines that affect people from different races in different ways. Someone could say that the MDs have a visual contact of the patient and, thus, they know their race. However, even then, it would be nice to have this data recorded for statistical purposes.”
P8, Nursing practitioner, Greece, Private	“I believe that, the ID number must be not only unique but, also, not the same as the Social Security Number (SSN) or personal insurance number or other. This can make it more secure and private. In that case, no patient would have to worry about whether his/her medical data is centrally recorded and accessed or not.”
P9, Lab/Pharmacist, Greece, Private	“It would be nice and helpful to include, somewhere and somehow, hereditary diseases.”
P10, ICT, Greece, Private	“In the demographics category data should be divided into three levels, i.e., basics, sensitive and administrative. In the sensitive level data like race and religion could be included if necessary. In the administrative level any data related to the medical unit and the patient could be included.”
P12, M.D., Greece, Private	“Occupation, Race, Religion are important since they are associated with certain diseases. Also, the education level and the language are important because they may assist in communicating with the patient.”
P13, Nursing practitioner, Greece, Private	“If we know the education level of the patient, then, the medical personnel will be able to communicate with the patient accordingly. However, I feel it is rather racist behavior to ask for it even for that reason.” “It is necessary to have the Race and the Nationality, especially nowadays, with all these immigrants and refugees moving from one country to another often with their diseases and not speaking our language. It so happens that, at the end of the day, the medical and nursing practitioners adapt to these people and not vice versa.”
P17, CEO/Admin, Greece, Public	“There is no need to have all the demographics in one level-view. Especially, the medical doctors don’t have enough time to see everything.” “In particular, as to the patient’s ID, it would be better off if each patient would have his/her own ID at a national level instead of a different one in every medical organization. There is an effort to do that now.” “As far as for the Race element, especially now that huge refuge waves are going to different countries including Greece, maybe it is appropriate to include it in the record.”
P16, Legal/Lawyer/QA, Greece, Private	“No: Name, Address, email, Race, Language, Size of family, Income level.” “The most important is not to have access to all this medical information to anyone.”
P18, M.D., Greece, Public	“No: Race, Income level, Language.” “There is no need and it is not good to ask for the income level. If there is an issue of whether the patient can cover his/her medical treatment and/or medication expenses or not the level of insurance coverage will indicate.” “In medication list it is suggested to have all the substances under one label e.g., Use of Substances.” “All categories should be able to accept additional entries (elements).”
P19, Nursing practitioner, Greece, Public	“Medicine and nursing are practices that are to be provided equally to everybody.” “It is possible that someone comes to the ER unconscious. In that case we will help him/her and provide everything necessary to save his/her life without looking into religious, race or other matters.” “For the category of Surgery, it would be better if it is by the biological systems for the human body.”
P30, Lab/Pharmacist, Oman, Public	“Blood group is important and must be included.”
P37, Legal/Lawyer/QA, Oman, Private	“Sometimes I feel that the medical organizations asked from me for more medical exams than necessary knowing my financial status. So, I believe Income level should not be included.”

**Table 2:** Economic feasibility—significant statements.

Alias, Position, Country, Hospital Type	Quotes
P5, Legal/Lawyer/QA, Greece, Public	“I don’t believe that the EMR will reduce the repetition of the medical examinations.”
P8, Nursing practitioner, Greece, Private	“It will help reduce costs and repetitions. Usually the expensive medical tests are the ones the results which don’t change quickly. So, there is no reason to ask between hospitals the same expensive tests very often.” “It’s everything for the patients and not for the profit. At the end some hospitals might lose profit, especially the private ones, but what is important is the quality of the patient diagnosis and treatment.” “This will make the MDs and the health professionals of all types, in private and public hospitals together, to cooperate for the benefit of the patient.”
P14, Lab/Pharmacist, Greece, Private	“The best health system is the one that a country can pay for. The problem is that you, also, have to pay for retirement plans, medical and pharmaceutical expenses, cancer treatment, heart disease treatment, economy growth, and more. Money is never enough for everyone for everything.”
P15, ICT, Greece, Private	“I believe a system like that will not reduce the repetition of medical exams but it will help to provide better health care.”
P17, CEO/Admin, Greece, Public	“It is important to implement the medical record system but not all medical organizations need to do so in the same depth. They need to study the economic feasibility first and then decide the depth.” “No need to fully deploy a medical record system if the cost is too high and the medical organization cannot bear it but start small and gradually move up. Make it using open architecture which means, implement the first part, check it and if everything works well then move to the next part.”
P18, M.D., Greece, Public	“In the case of Greece, and just for the case of the diabetic foot in which 250 million euros are spent every year, the savings will be enough to justify the cost for the implementation of the medical record management system”
P19, Nursing practitioner, Greece, Public	“It will cut the costs but it will increase the amount of work of the MDs. There are already complains of some MDs about the time they spend in entering data, even in simple similar programs, instead of practicing medicine. It is a big benefit but at the moment it is not practical especially if we want to record all the data, especially also given the economic crisis in the country.”
P20, Lab/Pharmacist, Greece, Public	“It needs an economic feasibility study to decide on the implementation of such as a system if it is too expensive. If we see it from the side of the patient the benefits are more.”
P22, Legal/Lawyer/QA, Greece, Public	“Is the implementation of such a system, regardless of the cost, a panacea? Not really.”
P28, M.D., Oman, Public	“A system like this will not save time, we will continue as doctors to spend the same time to treat a patient but what is important is it will help us not to forget anything important in the treatment. We will have everything in the system where we should report and update it. We are human beings and we forget some times.” “In a matter of health, we don’t talk about money. The most important is the health.”
P29, Nursing practitioner, Oman, Public	“Sometimes the patients are complaining about that the doctors are spending more time in front of the computer instead of examining them. The doctors have to do that because sometimes the patients are losing their medical reports or exams or even they are forgetting to inform the doctors about some of their own health issues. With a system like that a doctor can know always about the full patient’s history and they don’t have to do everything from the beginning. This can save patient’s life.”
P32, Legal/Lawyer/QA, Oman, Public	“When a country’s economy is in recession like it happens now in Oman and in other countries, it is very important to be able to cut the costs whatever you can. For example, if a person has made an X-ray recently there is no reason to ask to do it again. It costs money and affects their health since we know it may cause cancer. Similar with MRI.” “It will certainly save money. Maybe in the beginning it will cost a lot but if one thinks how much savings it will make just from the X-rays it is enough to understand how important it is.”

**Table 3:** Sharing medical records—significant statements.

Alias, Position, Country, Hospital Type	Quotes
P17, CEO/Admin, Greece, Public	“The medical exams belong to the patient since the patient had paid for them and s/he can do anything s/he wants with them. This is a practice applied not only in Greece but in Europe in general.” “It is necessary and wise to use the medical insurance coverage from patients from other European countries, since this way the Greek hospitals can benefit financially from charging these insurance companies instead of providing medical treatment and medication for free. This is especially important now that many foreigners come to Greece and many times they get medical services for free.”
P28, M.D., Oman, Public	“In some special cases with critical diseases, no matter if the patient doesn’t want to give to other doctors their personal medical information, as a doctor I am giving the information to save the patient’s life.”
P32, Legal/Lawyer/QA, Oman, Public	“It will be great to share medical information worldwide because we don’t share the patient but the patient’s data and this will help a lot to develop any research related to it and also make statistics based on it.”
P33, CEO/Admin, Oman, Private	“The Omani government is planning to implement a system that will allow any patient to have access to his medical record anywhere, anytime in Oman.”
P40, M.D., Oman, Private	“If it is properly secured is ok under authorization to exchange medical information.” “We trust people but we also mistrust them.”
